# Chemical sterilization with intratesticular administration of zinc gluconate in adult dogs: a preliminary report

**DOI:** 10.1186/s12610-019-0092-8

**Published:** 2019-08-01

**Authors:** Dorna Rafatmah, Asghar Mogheiseh, Davoud Eshghi

**Affiliations:** Department of Clinical Sciences, School of Veterinary Medicine, Shiraz University, P.O.Box 71441-69155, Shiraz, Fars Iran

**Keywords:** Dog, Histopathology, Testis, Testosterone, Zinc gluconate, Chiens, Histopathologie, Testicule, Testostérone, Gluconate de zinc

## Abstract

**Background:**

Chemical sterilization with zinc gluconate is being developed due to its permanent contraceptive effect in prepubertal dogs. In this study, five healthy adult dogs were selected randomly. Semen samples were collected and analyzed before the study to confirm normal testicular function. Under general anesthesia, pH neutralized zinc gluconate was injected directly into their testes. Testes diameter, ultrasonographic appearance, changes in the percentage of white blood cells, and testosterone concentration were monitored twice a week before and 1 month after the injection. At the end of the study, the dogs were castrated and their testes were removed for histopathological evaluation.

**Results:**

The general health of all dogs was normal after the injection. The appearance of testicular swelling was limited within 2 days of treatment. The average diameter of left and right testes was 2.48 and 2.03 cm before the injection and reached to diameter 2.12 and 2.15 cm, respectively, at the end of the study. Serum testosterone concentration was 4.2 ng/ml at the start and 4 ng/ml at the end of the study. The percentage of white blood cells at the start and end of the study were within normal ranges reported for dogs. Histopathological analyses indicate a degeneration of germ cells in seminiferous tubules, but Leydig cells retained their structure.

**Conclusions:**

Therefore, It is inferred that the injection of pH neutralized zinc gluconate into the adult dogs’ testes resulted in the loss of sperm-producing tissue without affecting the production of testosterone and the general health of adult dogs.

## Résumé

**Contexte:**

La stérilisation chimique par le gluconate de zinc a été développée en raison de son effet contraceptif permanent chez le chien prépubère. Dans la présente étude cinq chiens adultes en bonne santé ont été sélectionnés au hasard. Le sperme a été recueilli et analysé avant l’étude de façon à s’assurer d’une fonction testiculaire exocrine normale. Une solution de gluconate de zinc à pH neutre a été directement injectée dans les testicules sous anesthésie générale. Le diamètre des testicules et leur aspect échographique, les modifications des pourcentages de cellules blanches sanguines et la concentration sérique de testostérone ont été enregistrés deux fois une semaine avant l’injection et un mois après l’injection. Les chiens ont été castrés à la fin de l’étude et leurs testicules conservés pour analyse histopathologique.

**Résultats:**

L’état de santé général de tous les chiens fut normal après l’injection. Les testicules présentèrent un aspect gonflé qui resta limité aux deux premiers jours du traitement. Le diamètre moyen des testicules droit et gauche fut respectivement de 2,48 et 2,03 cm avant l’injection, et de 2,12 et 2,15 cm à la fin de l’étude. La concentration sérique de la testostérone fut de 4,2 ng/ml au début et de 4 ng/ml à la fin de l’étude. Le pourcentage de cellules blanches sanguines fut dans les fourchettes de la normale pour les chiens au début et à la fin de l’étude. L’analyse histopathologique a montré une dégénérescence des cellules germinales dans les tubes séminifères, mais la structure des cellules de Leydig était conservée.

**Conclusions:**

On peut par conséquent en déduire que l’injection de gluconate de zinc à pH neutre dans les testicules de chiens adultes entraine une perte du tissu à l’origine des spermatozoïdes sans affecter la production de testostérone et l’état général de santé des chiens adultes.

## Background

Both the uncontrolled populations and the large number of stray dogs are serious problems in many developing countries. The stray dogs` groups are a new risk for wild animals and may increase the risk of transmission of zoonotic diseases [1]. Approximately, 75% of dogs around the world, often referred to as poor-health free-roaming dogs, are uncontrolled; and this has led to the overpopulation of dogs [2]. According to the World Health Organization (WHO), an obligatory preventive public health action is canine birth control which should be permanently continued at the governmental organizations level as a supplementary action for control of zoonotic diseases [1]. Relative to the mass euthanasia and culling campaigns, surgical gonadectomy is the most commonly accepted method for controlling the population of dogs. However, due to many limiting factors, including anesthesia, equipment, adequate follow-up medical treatment, costs and risks of surgical procedures such as herniation, hemorrhage [3], and behavioral changes due to the reduction or deletion of the synthesis of steroids [4], surgical procedure may not be used as the sole method of controlling dog populations [5, 6]. For the last few years, several alternatives to surgical sterilization have been intensively investigated. There is a growing interest in non-surgical contraception, such as chemical sterilization, that causes the degeneration of testicles and destroys spermatogenesis, compared with surgical castration for controlling companion animal’s population growth [5, 7]. On one hand, an ideal chemical neutering agent would be one which is safe, affordable, effective, permanent, and a low-cost alternative, requires minimal facilities, poses minimal risk, could be delivered in a single injection [3, 8], and reduces androgen deprivation-dependent diseases [9]. However, androgen-dependent diseases, such as prostatic diseases and unfavorable behavior (urine marking, aggression, mounting), cannot be ignored [10].

A variety of chemical sterilants for neutralization have been used, but few products have been effective, safe or available for regular uses [11]. Researchers have used chemical agents for the castration of male dogs by intratesticular injection such as calcium chloride [3, 4, 8, 12], cadmium chloride [13], ferric chloride and ferrous sulfate [14], danazol [15], Bacillus Calmette Guerin (BCG) [15], glycerol [16], and lactic acid [17]. Among the common chemical sterilants available for chemical sterilization, zinc gluconate (with different brand names like Neutersol®/ Esterisol®) is considered a noncarcinogenic, nonteratogenic, and nonmutagenic material [18] and it can be used as a chemical sterilant agent in puppies [19]. Intratesticular treatment with an injectable zinc gluconate (neutralized in arginine) in dogs was first produced in the USA in 2003 and approved by the Food and Drug Administration (FDA) [20]. Zinc gluconate [21], has been injected into the testis, epididymides, and ductus deferens and it could cause infertility in male dogs by inducing testicular degeneration, causing irreversible disruption of spermatogenesis and azoospermia. It has also resulted in a reduction in the synthesis of testosterone, although this appears to be variable and may be affected by the dose administered, testicular size and the amount of testicular tissue destroyed [22–24]. The effect of zinc gluconate on puppies, aged up to 6 months old, has been studied in almost all of the reports. It has been estimated that about 2.5% develop adverse reactions [25]. Reported adverse effects included tissue necrosis, necrotizing orchitis and scrotal ulcerative dermatitis [5, 25]. The purpose of this study was to evaluate the effect of a single intratesticular zinc gluconate injection on testicular echotexture, histology, concentrations of testosterone, and inflammatory blood cells in adult male dogs (3 to 4 years-old).

## Material and methods

### Statement of animal rights

All studied animals and experimental study design were confirmed by the State Committee on Animal Ethics, Shiraz University, Shiraz, Iran (IACUC no: 4687/63). The recommendations of the European Council Directive (2010/63/EU) of September 22, 2010, about the standards in the safekeeping of animals used for empirical objects, were also followed.

### Animals and experimental groups

Five clinically healthy (without anatomical or reproductive disorders) adult mixed breed dogs (3 to 4 years old and 15–20 kg body weight) were selected. They were owned and kept by Shiraz University School of Veterinary Medicine. All the animals were housed individually in kennels and fed with standard dry food (300 g/dog/day; NUTRI™; Behintash Co. Iran) and water was provided ad libitum. They were kept under routine clinical observation for 30 days; and the breeding soundness evaluations, such as ultrasound examination of testes and prostate and sperm analysis were performed just before the start of experiment to ensure all dogs were in normal condition. Blood samples were collected from the jugular vein into plain evacuated tubes for determining concentration of testosterone and into tubes with EDTA for performing differential white blood cell counts in all dogs just before the treatment (day 0) and twice weekly for the next 37 days. Blood smears were prepared, fixed and stained with Giemsa for differential counting of white blood cells. Serum was harvested after centrifugation at 750×g for 10 min and stored at − 20 °C until evaluated for concentration of testosterone.

### Preparation and administration of zinc gluconate

In order to prepare neutralized zinc gluconate solution, 13.1 mg/ml zinc hydroxide (Titran, Iran) was dissolved in 1 ml of gluconic acid (Bayer, Germany), then it was neutralized to pH 7.0 by adding 0.2 M L-arginine (Merck KGaA, Germany) in glass vials, and filtered and sterilized by autoclave to be ready for injection.

**Fig. 1:**
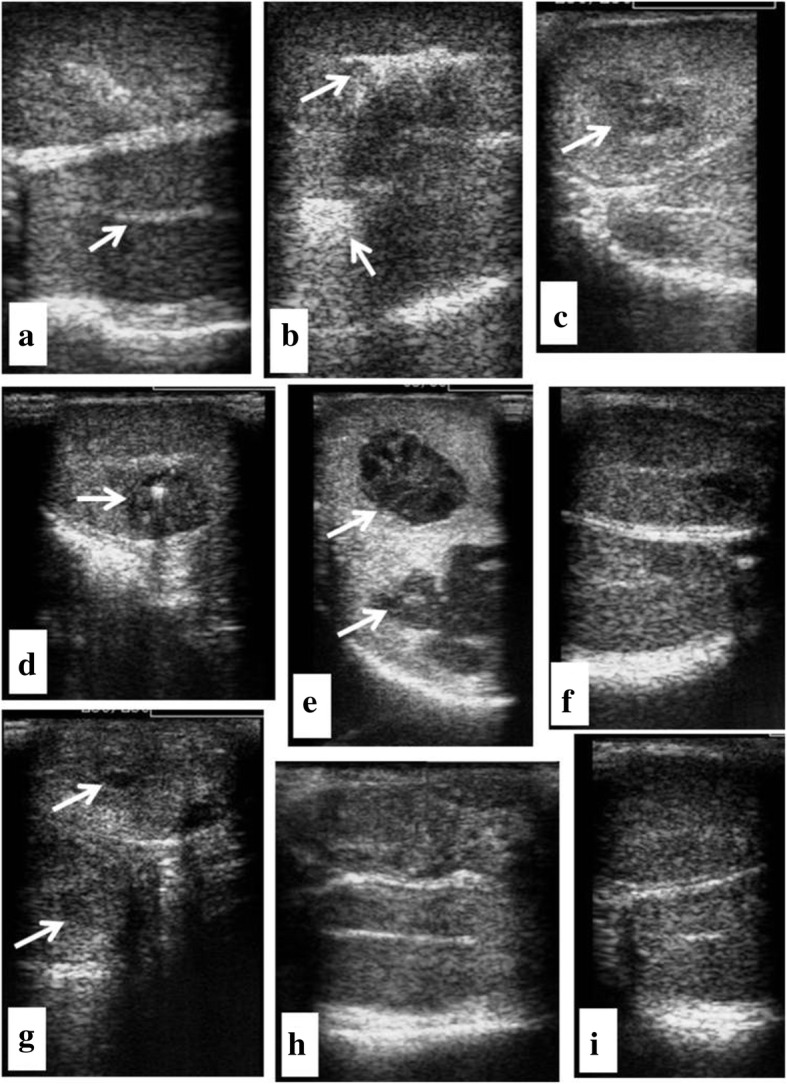
Ultrasonographic view of testes before (**a**) and 0 (**b**), 3, (**c**), 6 (**d**), 12 (**e**), 21 (**f**), 28 (**g**), 35 (**h**) and 37 (**i**) days after injection of zinc gluconate. **a** before injection, arrow indicates rete testis; **b** hyperechogenic echotexture indicates injected zinc gluconate solution (arrow); **c**, **d**, **e**, **g** arrows indicate formation and elimination of cystic like structures; **h**, **i** ultrasound view of the testes at the end of the study (day 37)

Sedation and anesthesia were induced with acepromazine 0.01 mg/kg and ketamine 10 mg/kg. Dogs were restrained in a dorsal recumbent position and the scrotum was disinfected with a 10% solution of Povidone iodine and alcohol. A single intratesticular dose of Zinc Gluconate was injected into each testis (24 G, 2.54 cm needle). The volume of injection was in accordance with the testis width [26]. The needle was inserted fully along the long axis of the testis, beginning near the head of the epididymis and the solution was carefully deposited while withdrawing the needle from the distal to the proximal of the testis. The deposited solution within testis was monitored by ultrasound examination (Fig. 1a, b).

### Morphologic and histological examinations

#### Ultrasound examination

The ultrasound examinations of testes were performed using a B-mode, ultrasound scanner (SIUI 800 V, China) equipped with a 7.5 MHz linear array transducer just before treatment and twice weekly for 37 days. Ultrasonography images were stored for the evaluation of inflammation, testicular diameter, and probable lesions.

#### Histopathology

One month after the intratesticular injections, all dogs were castrated under anesthesia and dissected testes were fixed in 10% buffered formalin. Five μm sections were prepared from the middle part of each testis, stained with hematoxylin and eosin (H&E), and evaluated with light microscopy.

### Statistical analysis

The results were analyzed using repeated one-way analysis of variance (ANOVA) using the statistical software package SPSS, version 16 (IBM Corp, Armonk, N.Y., USA). Data was presented as mean ± SEM. The statistical level was considered significant when *P*-value was less than 0.05.

## Results

The vital signs were recorded before and after treatment twice a day to be within normal limits. One of the dogs bit its left testicle, which caused a wound and led to the formation of abscess. After 2 weeks, the abscess ruptured and healed within 5 weeks. At the end of the study, both testicles were apparently normal and without inflammation. Testicular swelling was evident in two dogs by 1–2 days after the injection and, then, gradually reduced within 3 weeks after treatment. At day 37 post-injection, both testes were normal and without inflammation. In one of the dogs, an abscess-like structure was observed with ultrasound in both testes which subsequently reduced in diameter over time and was not detectable by the end of the study (Fig. 1c, d, e, g, h, i).

### Plasma testosterone concentration

**Fig. 2:**
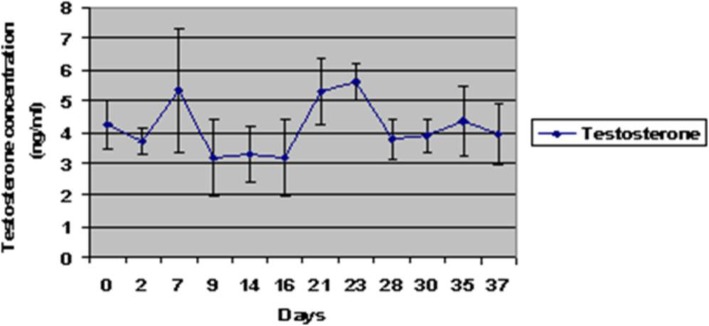
Changes in serum concentration of testosterone (mean ± SEM) of dogs following intratesticular injection of zinc gluconate. The testosterone levels maintained within physiological range at all time points

Concentrations of testosterone in serum of dogs did not significantly change over 37 days of the study following administration of zinc gluconate (Fig. 2). The mean concentration of testosterone was 4.25 ± 0.76 ng/ml at day 0, before the injection, and 3.93 ± 0.95 ng/ml at day 37 post-injection of zinc gluconate. During this period concentrations fluctuated between 3.18 ± 1.21 and 5.61 ± 0.57 ng/ml. The testosterone levels maintained within physiological range at all time points.

### Diameter of testes

**Fig. 3:**
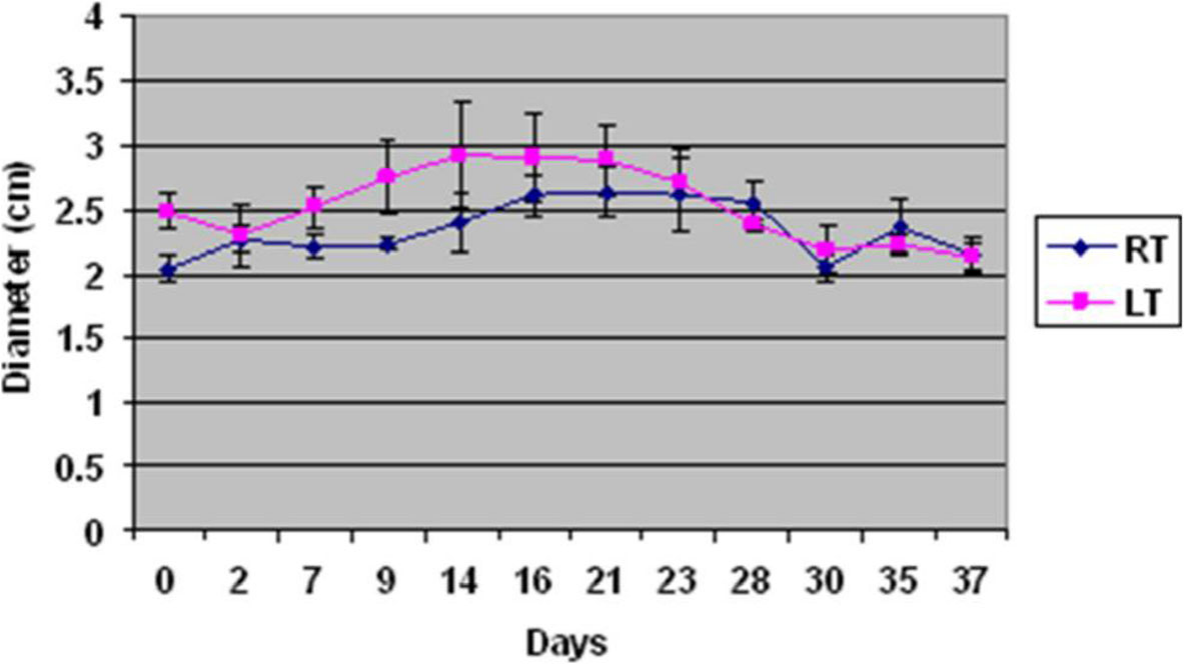
Testis diameter (mean ± SEM) of dogs following intratesticular injection of zinc gluconate (*n* = 4). Treated dogs revealed a reduction in testis width from day 21 to the end of the study

Changes of the mean diameter of testes (right and left) are presented in Fig. 3. The mean diameter of the right and left testis was 2.03 ± 0.1 and 2.48 ± 0.14 cm, respectively, at day 0, and 2.15 ± 0.13 and 2.12 ± 0.1 cm at day 37 of the study. The minimum and maximum mean diameter for testes were 2.03 ± 0.1 and 2.62 ± 0.19 cm (right testis) and 2.12 ± 0.1 and 2.92 ± 0.41 cm (left testis), respectively. Changes in the mean diameter of testes were not significant between different days of study, but it was significant between dogs (right testis *P* = 0.0003; left testis *P* = 0.006).

### Histopathology evaluation

**Fig. 4:**
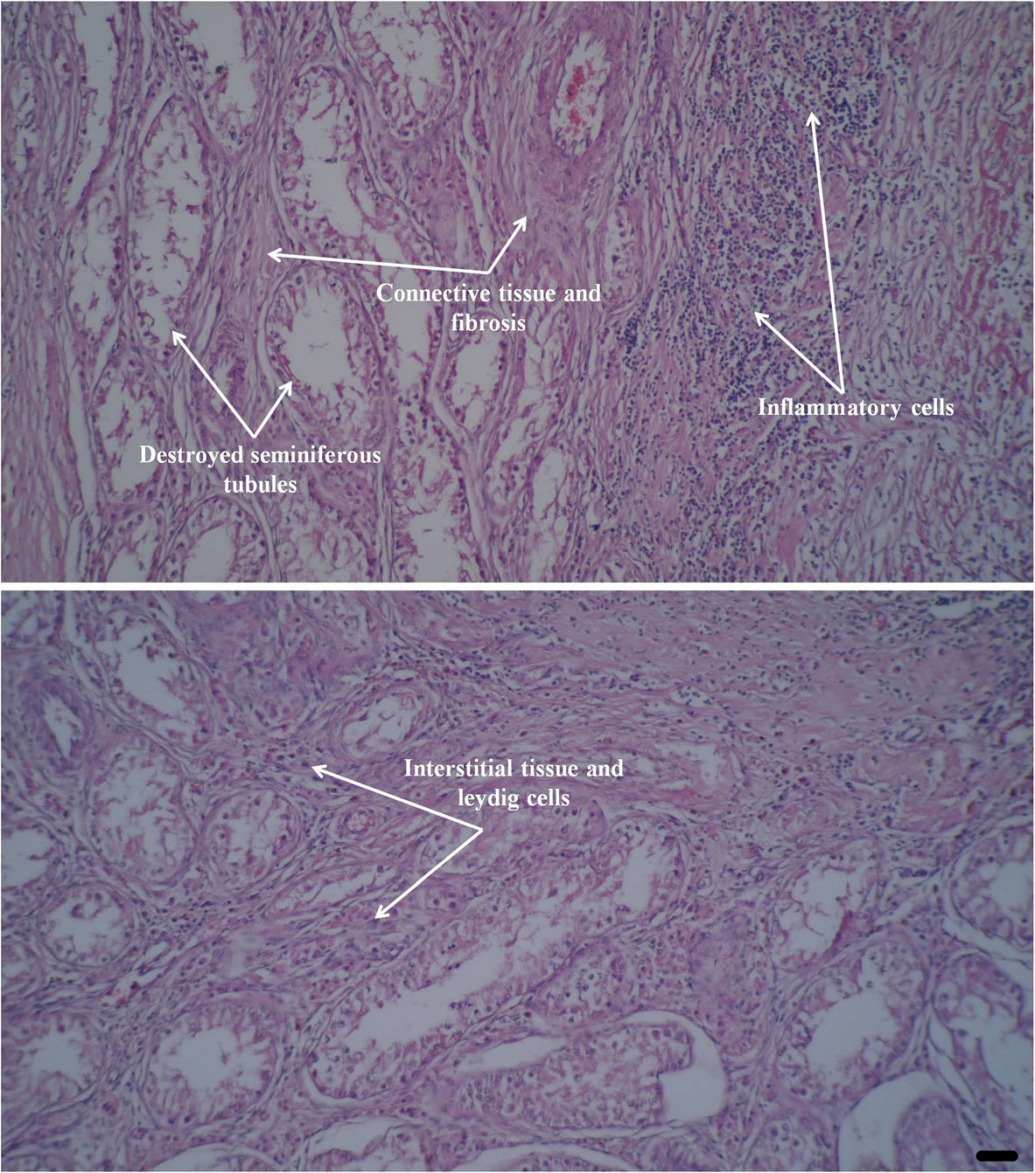
Histopathologic views of parenchyma of a dog’ testis presented 37 days after single intra-testicular injection of zinc gluconate. Degeneration of seminiferous tubules and accumulation of inflammatory cells were observed. In some parts, presence of connective tissue and fibrosis are evident 37 days after injection while Leydig cells and surrounding interstitial tissue are normal in appearance (H&E staining). Scale bar represents 13.8 μm

The histological examination of samples revealed the degeneration of spermatogenic tissues, the presence of fibrosis, inflammation, hemorrhage, and the presence of multinuclear spermatids. There was no sign of degeneration, necrosis, and fibrosis in the interstitial tissue and Leydig cells (Fig. 4).

### Inflammatory blood cells

The mean ± SEM percentage of the inflammatory blood cells presented before (day 0) and after the zinc gluconate injection in Table 1. Values are expressed in percentage of total white blood cells. There was no significant increase or decrease in the mean percentages of neutrophil, monocyte, basophil, and bands cells during the study. The mean percentage of lymphocytes was significantly different among dogs (*P* = 0.02). But changes in mean concentrations of lymphocytes over time were not significant. Also, significant differences were observed in the mean percentage of eosinophil between day 2 vs. 16 (*P* = 0.01), day 2 vs. 28 (*P* = 0.02), day 2 vs. 30 (*P* = 0.04), and day 9 vs. 30 (*P* = 0.02).

**Table 1:** The mean ± SEM percentage of the inflammatory blood cells presented before (day 0) and after the zinc gluconate injection. Values are expressed in percentage of total white blood cells

	Days of sampling											
Blood cells	0	2	7	9	14	16	21	23	28	30	35	37
Band cell	0.75 ± 0.25	0.25 ± 0.25	1 ± 0	0.75 ± 0.25	1.75 ± 0.62	1.75 ± 0.75	0.75 ± 0.47	1.75 ± 0.25	2.25 ± 0.47	1.25 ± 0.47	2.25 ± 0.75	0.25 ± 0.25
Eosinophil	2.25 ± 1.03	3.25 ± 0.47^a^	2.75 ± 0.62	2.75 ± 0.47^*^	0.75 ± 0.47	0.5 ± 0.28^b^	1 ± 1	1 ± 0.4	1 ± 0.4^b^	0.25 ± o.25^b*^	0 ± 0	0.25 ± 0.25
Lymphocyte	30.25 ± 5.93	28.5 ± 5.61	23.25 ± 1.10	22.5 ± 6.25	29.25 ± 5.25	15.25 ± 5.40	31.75 ± 4.69	28.75 ± 4.76	25.5 ± 5.51	34.75 ± 3.35	35 ± 5.49	31.75 ± 3.70
Monocyte	1.5 ± 0.64	1 ± 0.40	0.5 ± 0.5	0.5 ± 0.28	0.25 ± 0.25	1.25 ± 0.75	0.5 ± 0.28	0.5 ± 0.28	0.75 ± 0.47	0.5 ± 0.28	1 ± 0.40	0.5 ± 0.28
Neutrophil	65.25 ± 6.54	67 ± 5.47	72.25 ± 1.03	61 ± 7.72	68 ± 5.75	75.75 ± 3.27	66 ± 4.74	68 ± 4.70	70.5 ± 5.17	63.25 ± 3.19	61.75 ± 4.76	67.25 ± 3.42

^ab*:^Different superscript letter indicate significant difference in each row

## Discussion

The safety of zinc gluconate administration in dogs has been confirmed before [5, 7]. In this study the vital signs before and after treatment was recorded to be within normal limits. In other studies, such as the Cedillo et al. [27], similar results were observed and only 3.1% of dogs treated with zinc gluconate neutralized with arginine revealed ulcers or fistulas on the scrotum; other dogs showed a mild inflammatory response. Also, researchers observed an initial inflammatory response 15 days after the intratesticular injection of zinc gluconate as the acute phase of inflammation [27]. In most studies, mild testicular swelling and inflammation were reported to occur within a few days after treatment which usually resolved spontaneously [5, 7, 21, 28, 29]. Moreover, intratesticular administration of high concentrations of zinc caused an inflammatory reaction that was accompanied by the presence of macrophages, neutrophils, and predominantly CD8-positive lymphocytes [30]. In our study, systemic inflammatory response was not significant and significant difference was observed between dogs only in lymphocytes.

We did not find any significant change in the testosterone concentrations among dogs at the beginning and end of the study (37 days). Similarly, Vanderstichel et al. [26] did not observe any significant difference in circulating concentrations of testosterone between control and treated dogs between 4 and 6 months after the treatment. Also, in another study, the basal concentration of testosterone decreased initially, but after 2 years, it was not significantly different from untreated dogs [5]. Others [31] reported that testosterone concentration did not change in dogs after the first injection and during the experiment, but after the second administration of zinc gluconate, testosterone concentrations were lower than the normal range for the untreated male dogs in two different times of experiment (45 and 135 days). Oliveira et al. [29] reported a decline (40–60%) in the level of testosterone after the injection of a zinc-based solution, especially during the first 30 days, but differences were not significant between the treated and control dogs and the concentration of testosterone was in the normal range for dogs. These contradictory results could be due to differences in concentrations of testosterone among and within dogs, as well as differences in concentrations that can occur throughout the day, due to the pulsatile secretion of luteinising hormone in dogs which results in fluctuations in concentrations of testosterone [32]. In addition, variation in the number of zinc gluconate administrations, chemical composition of treatments, dose, testicular size, and age of dog could also have affected concentrations of testosterone after treatment [15, 26, 31].

Our findings also showed that there was no sign of regeneration in the Leydig cells at the end of the study (37 days) while others reported that the administration of zinc-based solution in the testis of dogs could cause necrosis, lipid degeneration, and death of Leydig cells 5 months after the treatment [25]. However, Vanderstichel et al. [26] observed a reproliferation and repopulation of Leydig cells after the testicular damage and that the testosterone levels would have increased later [31]. Moreover, unlike in surgical castration, testes were not removed following the intratesticular administration of zinc gluconate in dogs, so the source of testosterone was not eliminated completely [19].

Others indicated that libido in dogs was not significantly reduced following the injection of zinc gluconate in testes [29]. Many dog owners may prefer the preservation of some behaviors (e.g. guarding behavior) following gonadectomy. Administration of higher doses and repeated injections of zinc gluconate could preserve such behavior, but may cause some reduction in sexual aggression, mounting, libido, and spraying if concentrations of testosterone are reduced [31]. However, it is unlikely to completely suppress male-like behaviors such as roaming, sexual aggression, marking, or mounting as some elevation in concentrations of testosterone above basal concentrations remains likely [5, 6].

In this study, ultrasonography revealed normal structure and pathologic conditions such as abscess of testes. The use of ultrasonography for evaluation of echotexture, focal and diffuse anomalies and determination of testicular volume has been confirmed in dogs [33, 34]. So, it is a useful technique for monitoring changes that may occur following intratesticular injection.

## Conclusion

In conclusion, the injection of zinc gluconate in testes of adult dogs (3–4 years old) caused atrophy in the seminiferous tubules and disruption in spermatogenesis. It is inherently less invasive and more effective to the majority of dogs. Moreover, it is economical and can be easily performed. Careful attention should be paid to the administration technique so as to avoid undesirable side effects.

## Data Availability

Data and materials presented in materials and methods section.

## Abbreviations

FDA: Food and Drug Administration
WHO: World Health Organization

## References

[1] The reference revises as: World Health Organization. WHO expert consultation on rabies: second report: World Health Organization; 2013. https://apps.who.int/iris/bitstream/handle/10665/85346/9789240690943_eng.pdf?sequence=1&isAllowed=y. 24069724

[2] Icam coaliation. Humane Dog Population Management Guidance: 2007. Available from: http://www.icam-coalition.org/downloads/humane_dog_population_management_guidance_english.pdf.

[3] Jana K, Samanta PK (2007). Sterilization of male stray dogs with a single intratesticular injection of calcium chloride: a dose-dependent study. Contraception.

[4] Jana K, Samanta PK (2011). Clinical evaluation of non-surgical sterilization of male cats with single intra-testicular injection of calcium chloride. BMC Vet Res.

[5] Levy JK, Crawford PC, Appel LD, Clifford EL (2008). Comparison of intratesticular injection of zinc gluconate versus surgical castration to sterilize male dogs. Am J Vet Res.

[6] Massei G. Catch, inject and release: immunocontraception as alternative to culling and surgical sterilisation to control rabies in freeroaming dogs. In: Fooks A, Muller T, editors. Compendium of the OIE Global Conference on Rabies Control. Incheon-Seoul (Republic of Korea) OIE; 2012. p. 181–7.

[7] Soto F, Viana W, Mucciolo G (2009). Evaluation of efficacy and safety of zinc gluconate associated with dimethyl sulphoxide for sexually mature canine males chemical neutering. Reprod Domest Anim.

[8] Jana K, Samanta PK (2006). Evaluation of single intratesticular injection of calcium chloride for nonsurgical sterilization in adult albino rats. Contraception.

[9] Reichler I (2009). Gonadectomy in cats and dogs: a review of risks and benefits. Reprod Domest Anim.

[10] Bloomberg MS (1996). Surgical neutering and nonsurgical alternatives. J Am Vet Med Assoc.

[11] Fahim M, Wang M, Sutcu M, Fahim Z, Youngquist R (1993). Sterilization of dogs with intra-epididymal injection of zinc arginine. Contraception.

[12] Leoci R, Aiudi G, Cicirelli V (2019). Effects of intratesticular vs intraepididymal calcium chloride sterilant on testicular morphology and fertility in dogs. Theriogenology.

[13] Kar AB (1961). Chemical sterilization of male rhesus monkeys. Endocrinology.

[14] Kar AB, Kamboj VP, Goswami A (1965). Sterilization of male rhesus monkeys by iron salts. J Reprod Fertil.

[15] Dixit V, Lohiya N, Arya M, Argrawal M (1973). Chemical sterilization of male dogs after a single testicular injection of ‘Danazol’. Folia Biol.

[16] Wiebe JP, Barr KJ, Buckingham KD (1989). Sustained azoospermia in squirrel monkey, Saimiri sciureus, resulting from a single intratesticular glycerol injection. Contraception.

[17] Fordyce G, Beaman N, Laing A (1989). An evaluation of calf castration by intra-testicular injection of a lactic acid solution. Aust Vet J.

[18] Leonard A, Gerber G, Leonard F (1986). Mutagenicity, carcinogenicity and teratogenicity of zinc. Mutat Res.

[19] Kutzler M, Wood A (2006). Non-surgical methods of contraception and sterilization. Theriogenology.

[20] Alliance for Contraception in Cats & Dog. ZeuterinTM/EsterilsolTM, Product Profile and Position Paper. In: Dogs AfCiC, editor.: http://www.acc-d.org/docs/default-source/Resource-Library-Docs/pppp-zeuterinesterilsol-revised-may-2016.pdf?sfvrsn=2. In: ; 2016. p. 1–9.

[21] Oliveira EC, Moura MR, Silva VA (2007). Intratesticular injection of a zinc-based solution as a contraceptive for dogs. Theriogenology.

[22] Pineda M, Reimers T, Faulkner L, Hopwood M, Seidel JG (1977). Azoospermia in dogs induced by injection of sclerosing agents into the caudae of the epididymides. Am J Vet Res.

[23] Hopkins S, Schubert T, Hart B (1976). Castration of adult male dogs: effects on roaming, aggression, urine marking, and mounting. J Am Vet Med Assoc.

[24] Cavalieri J (2017). Chemical sterilisation of animals: a review of the use of zinc- and CaCl2 based solutions in male and female animals and factors likely to improve responses to treatment. Anim Reprod Sci.

[25] Forzán M, Garde E, Perez G, Vanderstichel R (2014). Necrosuppurative orchitis and scrotal necrotizing dermatitis following intratesticular administration of zinc gluconate neutralized with arginine (EsterilSol) in 2 mixed-breed dogs. Vet Pathol.

[26] Vanderstichel R, Forzan M, Perez G, Serpell J, Garde E (2015). Changes in blood testosterone concentrations after surgical and chemical sterilization of male free-roaming dogs in southern Chile. Theriogenology.

[27] Cedillo V, Vargas Pino F, Monroy O. Results of the massive sterilization project using gluconate neutralized with arginine in male dogs living in the States of Hidalgo, Mexico and Puebla. XVII Rabies in the Americas; Brasılia, Brazil RITA; 2016. p. 15–20.

[28] Wang M. Neutersol: intratesticular injection induces sterility in dogs. In: Proceedings of the International Symposium on Non-surgical Methods for Pet Population Control. Pine Mountain: ACC&D; 2002. p. 62–5.

[29] Oliveira EC, Moura MRP, de Sá MJ (2012). Permanent contraception of dogs induced with intratesticular injection of a zinc gluconate-based solution. Theriogenology.

[30] Leathem James H. (1977). Aging and the Testis. Advances in Physiology, Biochemistry, and Function.

[31] Vannucchi C, Angrimani D, Eyherabide A (2015). Effects of intratesticular administration of zinc gluconate and dimethyl sulfoxide on clinical, endocrinological, and reproductive parameters in dogs. Theriogenology.

[32] Thun R, Eggenberger E, Zerobin K (1990). 24-hour profiles of plasma cortisol and testosterone in the male dog: absence of circadian rhythmicity, seasonal influence and hormonal interrelationships. Reprod Domest Anim.

[33] England GCW (1991). Relationship between ultrasonographic appearance, testicular size, spermatozoal output and testicular lesions in the dog. J Small Anim Pract.

[34] de Souza MB, Silva LDM, Moxon R, Russo M, England GCW (2017). Ultrasonography of the prostate gland and testes in dogs. In Pract.

